# Surgical Outcome of Treating Grades II and III Meningiomas: A Report of 32 Cases

**DOI:** 10.1155/2013/706481

**Published:** 2012-11-05

**Authors:** Konstantinos Violaris, Vasileios Katsarides, Maria Karakyriou, Pavlos Sakellariou

**Affiliations:** ^1^Neurosurgery Department, Nicosia General Hospital, Nicosia, Cyprus; ^2^Neurosurgery Department, Interbalkan Hospital, Thessaloniki, Greece; ^3^Cardiology Department, Larnaca General Hospital, Larnaca, Cyprus; ^4^Neurosurgery Department, General Hospital “G. Papanikolaou”, Thessaloniki, Greece

## Abstract

*Aim*. To evaluate the frequency of atypical and malignant meningiomas and analyze recurrence rate; to study the morbidity and mortality of these tumors compared to benign meningiomas. *Methods*. During 1992–2007, 16 patients with malignant and 16 patients with atypical meningioma were operated in Neurosurgery Department of Thessaloniki's Papanikolaou Hospital. We analyzed tumor histology, location, and extent of surgical resection with respect to tumor reappearance and patients' outcome and compared the behavior of benign versus nonbenign meningiomas. *Results*. Malignant meningiomas accounted for 4.4% (16 patients) and atypical meningiomas for another 4.4% of the series of patients (353) who were operated for intracranial meningioma at our department that period. Malignant meningiomas recurred at a rate of 75% and atypical meningiomas recurred at a rate of 41.6%. There was a significant association of the histological classification (benign, atypical, and malignant) with recurrence (*P* < 0.01). The recurrence rate after complete resection was 13.8%. The recurrence rate for incomplete resection was 46.7%. Extent of tumor removal was significant to recurrence (*P* < 0.001) for benign as well for atypical and malignant meningiomas. Tumor location (*P* > 0.05) was not significant to recurrence. *Conclusions*. Atypical and malignant meningiomas appeared at a rate of 8.8% of our series of intracranial meningiomas. They showed a significant predisposition to recur. These rare subtypes have higher morbidity and mortality rates than benign meningiomas. Recurrence depends primarily on the extent of surgical removal and on the histological characterization of the tumor as atypical or malignant.

## 1. Introduction

A small percentage of intracranial meningiomas appear to have a malignant potential [1–3]. These rare histological subtypes characterized as malignant (grade III) and atypical (grade II) exhibit aggressive clinical behavior and are less studied than the classic benign (grade I) tumors [2, 4]. Objective of this study was to evaluate the incidence of atypical and malignant meningiomas and estimate their effect on recurrence, morbidity, and mortality. The postoperative behavior of the meningioma was followed and an effort was made to assess whether tumor's location, histopathological subtype, and the extent of surgical resection were predictive factors for recurrence. 

## 2. Clinical Material and Methods

From 1992 to 2007, thirty-two patients with grade II or III meningioma, were treated surgically by the staff of the Neurosurgical department of Thessaloniki “G. Papanikolaou” Hospital. This study is a part of an observational retrospective study that comprised 353 patients with intracranial meningiomas who were operated at our department that period. Hospitalization files, medical charts, and neuroradiological images obtained for the patients with meningioma, were analyzed with respect to clinical, operative, and laboratory pathological data. Tumor histological classification was conducted by the institute's pathologists. Postoperative follow-up examinations, carried out by the department's neurosurgeons were also used for this study. 

### 2.1. Follow-Up Examinations

The endpoint for recurrence was given by a computerized tomography scan (CT) or magnetic resonance image (MRI), showing a meningioma at a location contiguous with the previous surgery. The patients were brought in for follow-up examinations and neuroimaging controls at 3 and 6 months after surgery and then every year. They were evaluated via clinical examination or when that was not possible, by telephone interview. Living patients interviewed by telephone described their symptoms referable to brain tumor. For deceased, relatives supplied information and reported if death had occurred due to the tumor or by unrelated causes. The Karnofsky scale was used to evaluate the patients' outcome after the operation. 

### 2.2. Histopathological Study

 Tumors were divided into types based on World Health Organization criteria [5], with grade I subtypes characterized as meningothelial, fibrous, psammomatous, transitional, stroviloid, epithelioid, angiomatous microcytic, secretory, chordoid. grade II meningiomas are mentioned as atypical and grade III as malignant.

### 2.3. Completeness of Resection

 For evaluating resection, Simpson's scale of grading the extent of surgical removal was used [6]. This scale divides the extent of resection into 5 grades:

1. grade I: complete removal;
2. grade II: complete removal with coagulation of dural attachment;
3. grade III: complete removal, without coagulation of dural attachment or resection of involved sinus or hyperostotic bone;
4. grade IV: subtotal resection;
5. grade V: decompression biopsy.

For patients with resection grades IV and V, endpoint for recurrence was enlargement of the remaining tumor, shown on MRI or CT. 

### 2.4. Statistical Analysis

 The SPSS system (version 15.0.1) was used for statistical analysis of the experiment's results data. Analysisfor descriptive statistics for each variable was conducted. Quality controls for normality, means, and variances were also done.

## 3. Results

Meningiomas of histological grading II and III accounted for 8.8% of all tumors in our series (Table 1). The average patient's age was 49 ± 5 years at the time of surgery and the mean follow-up time was 4.3 years. Follow-up control for patients with nonbenign meningiomas revealed that grade III meningiomas recurred at a rate of 75% and grade II meningiomas at a rate of 41.6% (Table 1). Survival rates for three, five, and ten years for these subtypes were much lower than the rest of the meningioma patients (Table 2). Three-year survival rate was 66.6% for atypical meningiomas, 33.3% for malignant meningiomas, and 86.3% for patients with grade I meningiomas. Five-year survival rate was 58.3% for atypical and 8.3% for malignant meningiomas, while for benign cases it raised to 74.3%. Finally, ten-year survival rate was 33.3% for atypical and 0% for malignant meningiomas. On the contrary, a 66.7% 10-year survival rate was noticed for patients with benign meningioma. Six patients with malignant meningioma and two with atypical meningioma experienced tumor-related death. 

Complete tumor resection was accomplished in 20 patients (60%). The recurrence rate after complete resection was 40%. Patients with grade II resection (complete resection with coagulation of the dura) presented recurrence at 49% and grade III (complete resection of tumor, without coagulation of the dura or removal of affected sinus or bone) patients recurred at a percentage of 67%, while 100% of grade IV and V patients developed tumor enlargement. The extent of surgical tumor removal was significantly associated with recurrence (*P* < 0.001). Atypical and malignant meningiomas appeared to be more complex in resection than grade I tumors. Forty percent of them were characterized as gr. II–V in the Simpson scale, while the rate of nontotal tumor resection for the rest was 23.8%. 

More commonly, parasagittal (25%), convexity (18%), and tentorium (15%) tumors appeared in our series. Tumor location was not significantly associated with recurrence (*P* > 0.05).

Three patients with malignant meningiomas developed a metastasis. Metastases appeared at the parotid gland, the thoracic spinal cord, and a different site of the brain. The majority (72%) of recurrences were observed within two years from surgery and 96% within five years from surgery.

## 4. Discussion

According to some studies, malignant meningiomas comprise between 4.7 and 7.2% of meningiomas, whereas atypical meningiomas account for 1.0 to 2.8% [1, 3, 4]. The most known factor associated with their appearance is cranial irradiation [1, 6–8].

Though meningiomas are considered to be benign tumors, recurrence is observed frequently, with rates that vary between series [4, 8, 9]. The best accepted factor for prediction of recurrence is the 1957 Simpson grading system for completeness of resection [6], which evaluated invasion of the venous sinuses, tumor nodules in adjacent dura, and infiltration of unresected bone by meningothelial cells as chief causes for recurrence. The recurrence rates that Simpson refers to were 9% for grade I, 16% for grade II, 29% for grade III, 39% for grade IV, and 100% for grade V, respectively. In addition to that, some histological characteristics of malignancy favor recurrence. These are peritumoral brain edema [2, 10], increase of neovascularization [11], cellular pleomorphism, nuclear atypia, the presence of macronuclei, atypical mitoses, necrosis, and brain invasion [8, 12]. 

In our series, atypical and malignant meningiomas accounted for 8.8% of all cases (4.4% each). Recurrence was observed at a rate of 41.6% for grade II tumors. For grade III meningiomas, recurrence rate was 75%. Besides the histological “aggressive” features we also found that recurrence was significantly associated with resection extent, according to Simpson grading system. Tumor location was not significantly related with meningioma reappearance, with the exception of certain locations whereas total resection was impossible. 

The extent of tumor surgical removal was the most important factor for recurrence.

The rate of recurrence diminishes with time from operation. Within 5 years from surgery, 96% of tumor reappearances were observed. 

Three patients (37.5%) of the patients with malignant meningiomas developed a metastasis (parotid gland, thoracic spinal cord, and a different site of the brain). This shows that there is a high tendency of these tumors to develop metastases. 

The role of radiotherapy is well established in the treatment of atypical and malignant meningiomas [1, 13–16]. Patients with these types of tumor are sent for radiotherapy postoperatively, if total resection is not possible. This is more frequent at tumor locations with difficult or high-risk access, where the surgeon tends to be less aggressive. Conventional external beam radiation is being used for years and stereotactic radiosurgery is reported for unresectable tumors [3, 17]. Improved rates of survival for patients with malignant meningioma are mentioned after radiotherapy [13]. In addition to that, lower recurrence rates were observed in patients undergoing immediate postoperative radiation [16]. Another study supports that any tumor remnant radiologically demonstrated on postoperative imaging should be treated with radiosurgery and that postoperative radiotherapy after a first-time resection should be reserved for tumor remnants too large for radiosurgery and for which a second-stage operation is not planned [15].

In our study, five patients with grades II and III meningiomas were sent for stereotactic radiotherapy after incomplete tumor resection. Further regression of the tumor was reported (by the treating radiotherapists) in three of them.

Another important issue to be cleared is whether meningiomas sometimes progress histopathologically to a higher grade and develop aggressiveness after they are operated. Some series have shown that up to 2% of all benign meningiomas transform into malignant [18]. This is supported by other studies also [3, 7], but other writers reject it [12]. In our series, we had one patient with grade I meningioma which progressed to grade II according to the histopathological result of the second resection.

## 5. Conclusions

Atypical and malignant meningiomas appear to be distinct entities with poor prognosis, despite the surgical intervention. Radical tumor excision is the most effective treatment, as it determines the patient outcome, and it should always be applied. In cases of subtotal resection, radiotherapy should be applied, as it seems to delay tumor's reappearance.

## Figures and Tables

**Table 1:** Histology and recurrence.

Tumor histologic subtype	Number of patients	Percentage of recurrence
Atypical (gr. II)	16 (4.4%)	41.6%
Malignant (gr. III)	16 (4.4%)	75%
Benign (gr. I)	321 (91.2%)	21.5%

**Table 2:** Malignant meningiomas and outcome.

Tumor subtype	3-year survival	5-year survival	10-year survival
Atypical	66.6%	58.3%	33.3%
Malignant	33.3%	8.3%	0%
Benign (gr. I)	86.3%	74.3%	66.7%

## Abbreviations

CT: Computed tomography
MRI: Magnetic resonance imaging.
